# Supplementary material for: Development of Palatable Amorphous Trazodone Hydrochloride Formulations via Ion Exchange
Source: Pharmaceutics. 2025 Jul 27;17(8):972. doi: 10.3390/pharmaceutics17080972 (PMC12389288; doi:10.3390/pharmaceutics17080972)
Supplement: Supplementary file 1 [file pharmaceutics-17-00972-s001.zip › pharmaceutics-3747160-supplementary.pdf]

The effects of resin type on the ion-exchange process:

Table. S1 Formulation design of TRCs (resin type).

Formulation	TRA (mg)	Amberlite IRP69 (mg)	Amberlite IRP88 (mg)	Water (mL)
F1	300	300	/	600
F2	300	/	300	600

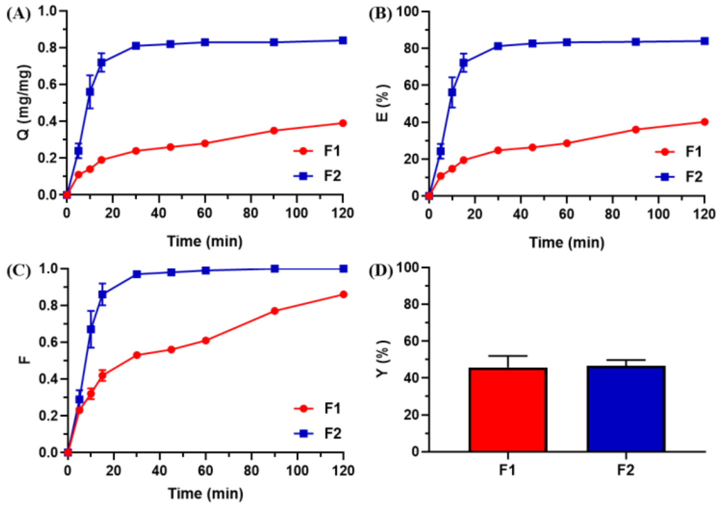

Figure. S1 Effects of different types of Amberlite on (A) drug loading, (B) drug utilization, (C) degree of drug loading, and (D) yield. (mean±SD, n=3).

Amberlite IRP88 has higher drug loading, drug utilization, and faster reaction rate than Amberlite IRP69. Therefore, Amberlite IRP88 was selected for the preparation of TRA resin complexes.

The effects of solvent on the ion-exchange process:

Table. S2 Formulation design of TRCs (solvent).

Formulation	TRA (mg)	Amberlite IRP88 (mg)	Water (mL)	Ethanol (mL)
F3	300	300	600	/
F4	300	300	300	300
F5	300	300	/	600

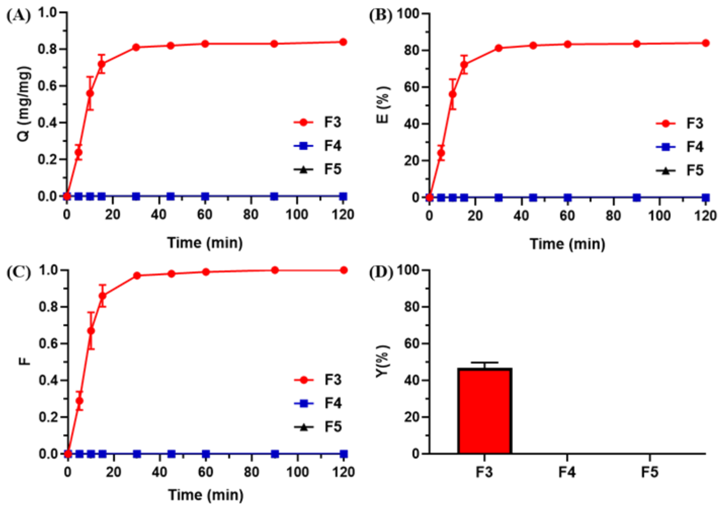

Figure. S2 Effects of different solvents on (A) drug loading, (B) drug utilization, (C) degree of drug loading, and (D) yield. (mean±SD, n=3).

Amberlite IRP88 and TRA can form TRCs in water, but were not capable of ion exchange in solvents containing ethanol.

The effects of drug concentration on the ion-exchange process:

Table. S3 Formulation design of TRCs (drug concentration).

Formulation	TRA (mg)	IERs (mg)	Water (mL)
F6	100	100	1000
F7	300	300	600
F8	300	300	300

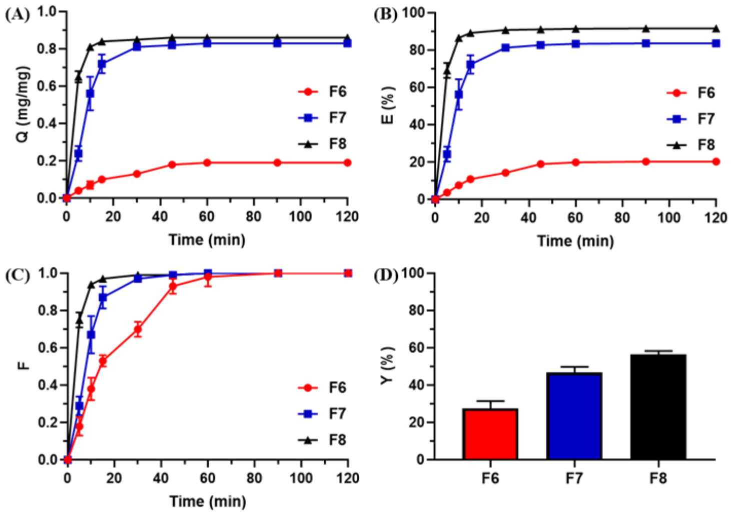

Figure. S3 Effects of TRA concentration on (A) drug loading, (B) drug utilization, (C) degree of drug loading, and (D) yield. (mean±SD, n=3).

The higher the initial drug concentration, the higher the drug loading, drug utilization, and yield of TRCs. Therefore, the drug concentration of 1.0 mg/mL was the most suitable.

The effects of the pH of solvents on the ion-exchange process:

Table. S4 Formulation design of TRCs (the pH of the solvent).

Formulation	TRA (mg)	IERs (mg)	Water (mL)	pH 1.0 (mL)	pH 4.5 (mL)
F12	300	150	300	/	/
F13	300	150	/	300	/
F14	300	150	/	/	300

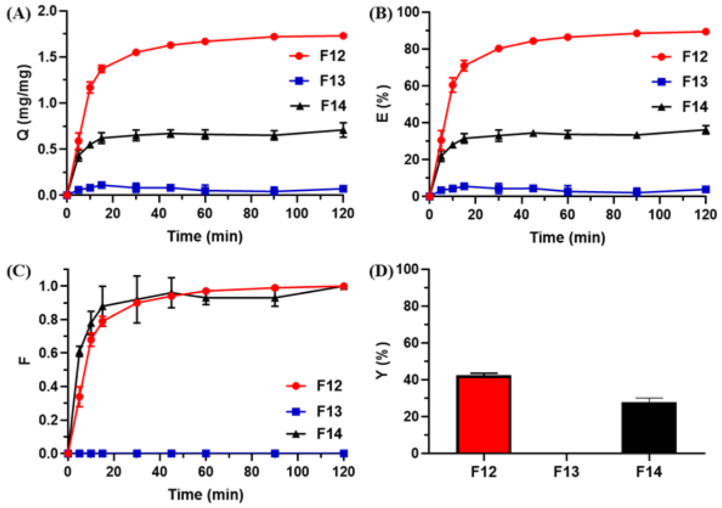

Figure. S4 Effects of pH of solvents on (A) drug loading, (B) drug utilization, (C) degree of drug loading, and (D) yield. (mean±SD, n=3).

As the pH of the solvent decreases, the drug loading and drug utilization decrease. Therefore, pure water was the best reaction solvent.

The effects of stirring speed on the ion-exchange process:

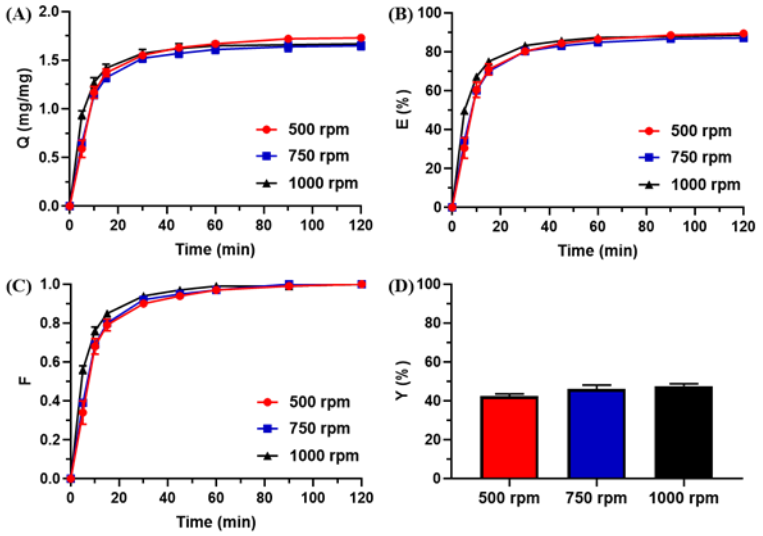

Figure. S5 Effects of stirring speed on (A) drug loading, (B) drug utilization, (C) degree of drug loading, and (D) yield. (mean±SD, n=3).

The results suggested that stirring speed had no effect on the ion exchange process.

The effects of temperature on the ion-exchange process:

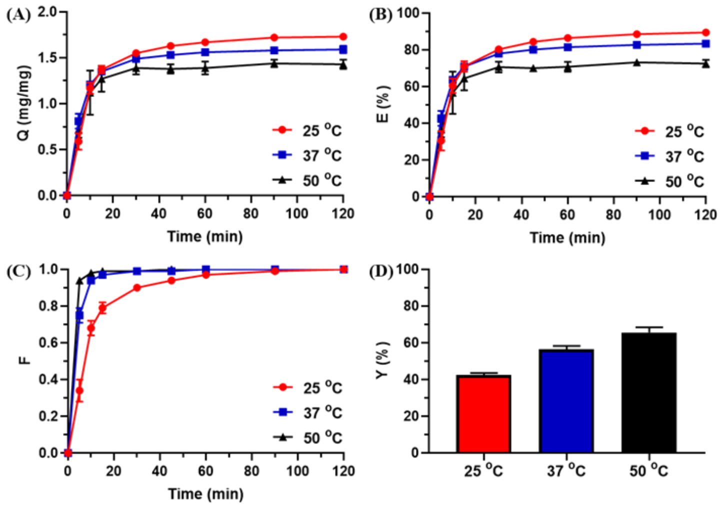

Figure. S6 Effects of temperature on (A) drug loading, (B) drug utilization, (C) degree of drug loading, and (D) yield. (mean±SD, n=3).

The improvement of both the degree of drug loading and yield indicated that increased preparation temperatures could promote the ion exchange process.

Therefore, 50 ℃ was selected as the optimal preparation temperature for the preparation of TRCs.
